# Multi-criteria analysis in the health area: selection of the most appropriate triage system for the emergency care units in natal

**DOI:** 10.1186/s12911-020-1054-y

**Published:** 2020-02-21

**Authors:** Deyse Gillyane Gomes Camilo, Ricardo Pires de Souza, Talita Dias Chagas Frazão, João Florêncio da Costa Junior

**Affiliations:** Universidade Federal do Rio Grande do Norte, Natal, Brazil

**Keywords:** Multicriteria decision analysis, FITradeoff, Emergency care units, Triage system

## Abstract

**Background:**

Multiobjective decision-making processes present a high degree of complexity in their solution, and tools such as multicriteria decision analysis appear as a way to facilitate the decision-makers’ solution and ensure that the decision is made cohesively and efficiently. In the public health sector, decisions are even more delicate because they work not only with the direct influence of human needs, but also with limited financial resources. An important point for the emergency care units is the triage system, which consists of a pre-evaluation of the patients, classifying them according to the degree of life risk. Through triage, the patient can be attended more quickly and efficiently, streamlining the whole process. Thus, the present research endeavored to determine the most appropriate triage protocol for emergency healthcare units in Natal-RN city in Brazil and may help others less advanced countries to determine the most appropriate triage protocol for emergency healthcare.

**Methods:**

In this study, we used the multicriteria analysis method known as FITradeoff. In addition, interviews and structured questionnaires applied with nurses, specialists and directors.

**Results:**

Based on the questionnaires and preferences presented by the decision-makers, the Spanish Triage System was the most suitable protocol for the emergency care units, which presented with high ease of use and implementation.

**Conclusions:**

This study reached its main objective, which was to determine the most appropriate triage protocol. In addition, it was observed the possibility of new research, such as the development of a specific protocol for this emergency care units and the creation of an application software for this new protocol.

## Background

Brazil is known to have a structurally differentiated public health system that endeavors to promote a complete and unrestricted care system to the population. However, this bold objective is far from being accomplished adequately, because the healthcare system has faced several constraints, such as investment cuts, congested processes and an overwhelming demand. Thus, it is important to discuss new ways of improving the healthcare system, at the same time as dealing with the investment reductions [1–3].

Among the programs promoted by the Brazilian Unified Health System (SUS), there are the Emergency Care Units (UPA), which are fixed units for urgent and emergency care, with the purpose of attending to cases of medium and low complexity in order to support large hospitals in cities [4].

The most diverse types of patients presenting the most diverse symptoms can be attended by the UPA, they will receive a specific treatment to the presented problem. However, what will be shared by everyone will be the service triage process.

In the triage system, patients should be evaluated according to a previously defined protocol, and will be classified according to the degree of urgency of care. In this way, it is possible to treat the most serious patients first, ensuring that everyone has a treatment of excellence [5].

Around the world there are several types of triage protocols known for their quality and efficiency, as well as their characteristics that differentiate them and make them more appropriate for a given type of healthcare center, according to the characteristic of each type of center will present a more appropriate triage protocol to be used [6].

In order to select the most appropriate triage protocol for the emergency care unit, it is important to make a comparison between the protocols, and to know well the characteristics of the place to be implemented. However, in order to make this decision the manager finds it very difficult, because the problem has several variables to analyzed, making the decision model very complex.

Thus, there are tools to assist the decision-making process, such as the Multicriteria Decision Analysis (MCDA), which consists of analyzing the criteria for the choosing, where a group of specialists or scholars to give notes to the criteria. In this way, it is possible to evaluate the possible solutions and determine which is best for a given problem.

The city on the study has four UPAs and all were included in the research; furthermore, those UPAs do not have any structured protocol set in their units, causing bottlenecks and screening breakdowns, rendering the overall health system inefficient.

Thus, the present study aims to determine the most appropriate triage protocol to be used in the Emergency Care Units in Natal-Brazil, using the FITradeoff multicriteria method. This is in the face of the perception presented by the nurses and the unit directors. On the whole, the present study proposes a new application of multicriteria decision analysis in healthcare systems; for no research using MCDA was found to assist the decision making process of choosing the best screening protocol.

It is noteworthy that studies like the present one – with little or no monetary investment for research, carried out in small units with an excess demand – represent a very common situation in the poorest and most disadvantaged countries, thus increasing its replicability and usefulness.

## Methods

### Brazilian health system

The concept of health is established by the union of the political, economic and socio-cultural systems of a nation, based on individual values and the collective demands of society. Thus, each place and time will have a different concept for ‘health’ based on the structure of society and the vision of the population [7].

The structure of Brazilian public health is based largely on the political, social and economic relations of the country through time, wherein it is possible to observe the organization according these tendencies of society.

Brazil’s national public health policy began to take shape from the twentieth century onwards with the organization of sanitary practices in Brazil, supported by the sociopolitical context of Brazilian capitalism. Public health is an integration between public responsibility and social law [8–10].

The Brazilian Unified Health System (SUS) started to develop and grow as a management system in Brazil from 1990, where the union is the main financier of SUS services, and it is responsible for creating national public policies for health. Therefore, the SUS creates programs and national actions, which must be accepted and implemented by states and municipalities [9, 11].

Within this series of programs created, there is the National Politic of Attention to Emergencies and Urgencies (PNAU) that aims to regulate actions directed at the public of emergency care in Brazil, PNAU is composed of eight programs of action, through different levels of care. They control and direct urgency and emergency health services [12].

Among these, there is an important program that is the Emergency Care Units (UPA), which are care units to attend small damages, avoiding overcrowding in large hospitals [8].

### Emergency care units (UPA)

The Emergency Care Units (UPAs) are very important within the emergency care system, since they increase service coverage to population, based on the territorial dimension of the country. In addition, in contrast to the Brazilian health system that presents many deficits in care, UPAs present themselves as a solution to the great demand for medium complexity care [13].

An important sector to analyze within the UPAs is the host system provided by these institutions. The host is a form of humanization of care, that is, care is performed responsibly, with commitment to listen and value the patient, creating an environment of trust between both parties, and working with a first contact of the health services [14].

The patient’s triage process emerged within the host system. It has risen from the need to organize and classify urgency and emergency services, directing the patients in the best way possible [13, 14]..

### Triage system

The word triage comes from the French ‘trier’, which means to choose, to select. Triage has always been widely used by man, but his study stands out for its use in war strategies, giving support in the planning of wars [6].

The emergency triage system has emerged with the need for some more developed countries, such as Australia and Canada, to reduce the overcrowding of this type of care and to adjust their health care system to patient demand and need with available resources, ensuring efficient system management [15].

These countries developed the risk classification system in order to assess the patient’s level of urgency and the average waiting time for the care, thus making it possible to refer patients more quickly to the appropriate care area [16].

Around the world there are several types of protocols of risk classification, it is important for the present research to know the characteristics and specifications that differentiate them.

### Triage protocols

Nowadays it is increasingly recommended by health councils around the world that health systems use the already consolidated host protocols, with the intention of guaranteeing reliability, trustworthiness, and validation for the triage performed [17, 18].

**Fig. 1:**
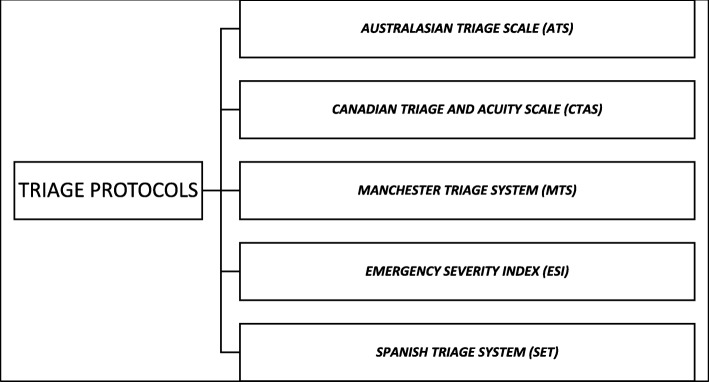
The top five triage protocols

There are five triage protocols established and globally recognized: the Manchester Triage System (MTS), the Australian Triage Scale (ATS), the Canadian Triage Acuity Scale (CTAS), the Emergency Severity Index (ESI), and the Spanish Triage System (SET) (Fig. 1) [19, 20].

For this research, these five pre-established protocols will be analyzed and structured, to the point of indicating their main characteristics and their criteria of evaluation and classification of the risk of the patient.

Thus, Table 1 was put together, showing the relationship between the protocols studied and the criteria and subcriteria used in the research. In order to develop the table, the present paper used several researches and studies on the five protocols (Fig. 1), as to find out if they bring out concepts and insights on the subcriteria and criteria used in the present research [20–33]..

**Table 1:** Relationship between alternative vs. subcriteria

Criteria	Subcriteria	ATS	CTAS	MTS	ESI	SET
Guidelines	Adult	1	1	1	1	1
	Pediatric	1	1	0,5	1	0
	Senior	1	0	0,5	0	0
	Pregnant	1	0,5	0	1	0
	Disabled	0	0,5	0,5	0	0
	Aggressive	1	0	0,5	0	0
	Alcoholics	0	0	0,5	0	0
	Nurses Attendance in Simple Cases	0	0	0	0	1
Ease of Evaluation	Medical History	1	1	1	1	1
	Pain Scale.	1	1	1	0	1
	Use of Medications	0	1	0	0	0
	Allergies	0	1	0	0	0
	Physical Evaluation	1	1	0	0	0
	Mental Evaluation	1	0	0	0	0
	Vital Signs	0,5	0	0	0	1
	Re-triage	1	1	0	0	0
Ease of Use	Use of Color Scale	1	1	1	1	1
	Maximum Waiting Time	1	1	0	0	0
	Maximum Triage Time	1	1	0	0	0
	Maximum Service Time	1	1	1	0	1
	Total Triage (5 levels)	1	0	1	0	1
	Partial Triage (2 + 3 levels)	0	1	0	1	0
	Nurses Attendance in Simple Cases	0	0	0	0	1
	Use of Computer and Software	0	0,5	1	0	1
Ease of Implementation	Training of employees	1	1	1	1	1
	Need for computer	0	1	1	0	1
	Use of specific software	0	0	1	0	1

Where:

- 1 ➔ It has satisfactory citation on the subject;
- 0 ➔ I has not citation on the subject;
- 0,5 ➔ It has few citations on the subject.

Table 1 consists of four criteria: (a) Guideline evaluates the presence of treatment and evaluation instructions for different types of patients; (b) Ease of Evaluation assesses the presence of indicators that facilitate a patient assessment by nurses; (c) Ease of Use evaluates characteristics that will facilitate the use and control of protocols in the daily routine of the units; and (d) Ease of Implementation assesses how simple a protocol implementation will be according to the characteristics of the units.

### Multicriteria decision analysis (MCDA)

The Multicriteria Decision Analysis emerged in the early 1940s, when several scholars began to worry about how to rationalize the decision-making process, even if indirectly contributing to the conception of this new tool [34].

From the 1960s, the first probabilistic decision-making methods emerged, based on a less complex mathematics, but still correct and adequate for the procedure. Nevertheless, it was from the 1970s that the multicriteria analysis began to strengthen itself, supported by a scientific community increasingly concerned with this type of research [34, 35].

Decision makers tend to take over its management several decisions, these are often the comparison process, which classify and order your options. For similar problems, different decision makers can take different solutions, since these can assign different values to the analysis criteria [35, 36].

In addition, the decision system based on the multicriteria analysis aims to support the decision recommending actions or directing these actions, but not indicating a specific solution to the problem analyzed, since this can be modified according to the will of the final decision maker [37].

### FITTRADEOFF

The method of multi-criteria decision analysis FITradeoff is a model developed in the year 2016 by researchers from the Federal University of Pernambuco, Brazil, and is a result of modernization of world-known method Tradeoff [38, 39].

The traditional Tradeoff method is known for its complex process of comparison between criteria, which makes it very difficult for the decision-maker to work, since the decision maker needs to give exact values of the peer evaluation, demanding a high degree of knowledge about all the evaluated points and hindering a fully conscious choice [38].

In this way, FITradeoff emerges as a flexible and interactive method that significantly reduces the cognitive effort used in the choice and decreases the amount of information needed by the decision maker [38, 40].

The FITradeoff was developed with the purpose of creating a decision support model that was more accessible and simple in the eyes of the decision maker, and which minimized the complexity when weighting the criteria and the process of evaluating the alternatives [41, 42].

The method in question is classified as an additive model, which performs comparisons pairwise between the solutions, and using tradeoffs system as a basis, guaranteeing a compensation among the criteria [41].

In FITradeoff, as well as in many additive MCDA methods, the term weights are not used, but rather scale constants, which consists of an approximation of the value and that facilitates the application of the model to the decision maker [38, 43].

Thus, to work with the evaluation process of the criteria we have [43, 44]:

1 $$ \upsilon (x)=\sum \limits_{i=1}^n{k}_i\ {\upsilon}_i\ \left({x}_i\right) $$

Assuming that:

2 $$ \sum \limits_{i=1}^n{k}_i=1 $$

3 $$ {k}_i\ge 0 $$

Where:

x = Consequences of each alternative;

i = Criteria;

k = Scale Constant (Weight);

*υ*_*i*_ (*x*_*i*_) = Relative function of the consequences for each criteria.

Starting from the application process of the FITradeoff model, it can be assumed that it is subdivided into two stages, as well as the traditional Tradeoff, first obtaining the scale constant of each criterion ‘ *k*_*i*_ ’ using the preference ‘P’, and second obtaining the values ‘ *k*_*i*_ ’ using the indifference relation ‘I’ [44].

In the first step the weight space is given from the preferences of the decision maker on the criteria, that is given directly in the software, and the previously determined scale constant data [41, 44].

4 $$ {\varphi}_n=\left\{\left({k}_1,{k}_2,{k}_3,\dots, {k}_n\right)|{k}_1>{k}_2>{k}_3>\dots >{k}_n;\sum \limits_{i=1}^n{k}_i=1;{k}_i\ge 0\right\} $$

In the second step, if one has what distinguishes the FITradeoff from the traditional Tradeoff, the decision maker does not need to specifically determine the value of the weight to the criteria, he simply inserts the upper and lower limits, and from the preferences of the decision maker himself, limits are reduced until reaching the solution [38, 41].

5 $$ {\upsilon}_i\ \left({x}_i^{\prime}\right)>\frac{k_{i+1}}{k_i} $$

6 $$ {\upsilon}_i\ \left({x}_i^{\prime \prime}\right)<\frac{k_{i+1}}{k_i} $$

Where:

$$ {x}_i^{\prime } $$ = Maximun limit;

$$ {x}_i^{\prime \prime } $$ = Minimun limit.

In this way, a new space of weights ‘ ${\varphi}_n^S$ ’ will be developed that will respect all the restrictions, and that will be part of the previous space of weights [44].

7 $$ {\varphi}_n^S=\left\{\begin{array}{c}\left({k}_1,{k}_2,{k}_3,\dots, {k}_n\right)/{k}_1>{k}_2>{k}_3>\dots >{k}_n;\sum \limits_{i=1}^n{k}_i=1;{k}_i\ge 0\\ {}{k}_1\ {\upsilon}_1\ \left({x}_1^{\prime \prime}\right)<{k}_2<{k}_1\ {\upsilon}_1\ \left({x}_1^{\prime}\right);\dots; \\ {}.\\ {}.\\ {}.\\ {}{k}_{n-1}\ {\upsilon}_1\ \left({x}_{n-1}^{\prime \prime}\right)<{k}_n<{k}_{n-1}\ {\upsilon}_1\ \left({x}_{n-1}^{\prime}\right);\dots; \end{array}\right\} $$

This new space created (${\varphi}_n^S$) will be presented in the form of lower and upper limits, which will be reduced to the point of including only a final solution, which is the best possible solution according to the preferences presented by the decision maker [38, 41].

### Methodological process

**Fig. 2:**
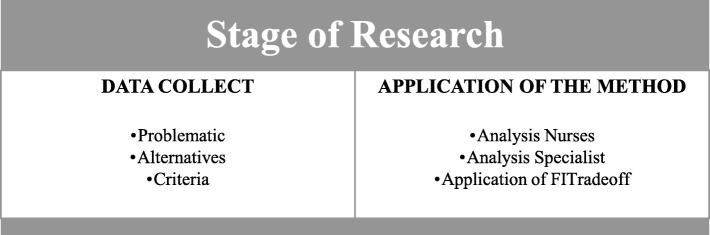
Stages of Research

The methodological process of this research can be better understood if it is separated into two main steps: First, the data collection process and, secondly, the FITradeoff application (Fig. 2).

In Fig. 2, the data collection process can be described as all stages common to the application processes of a multicriteria method. The FITradeoff Application stage consists of all the specific steps for this study.

I. FIRST STEP

The first stage starts following the MCDA application parameters presented by [34], which consists of three basic steps: Definition of the problem, Definition of the alternatives and Definition of the criteria [45, 46].

1. For the problem definition, the main objective of the research and the desire of the final decision maker were taken as the basis, and this was classified as a choice problem.
2. In the definition of the alternative, we used the lifting process by studying the literature, where it was sought to identify and know the main screening protocols known and disseminated in the world.
3. The definition of the criteria is based on structural and behavioral characteristics of each of the protocols selected in the previous step. Thus, criteria and subcriteria were developed, which facilitated subsequent evaluations. In addition, the criteria went through a process of validation with the final decision maker, which evaluated the relevance of the criteria and subcriteria for the purpose of the study.

II. SECOND STEP

After obtaining the basic data for the application common to any multicriteria method, we start the second step, which is a more applied phase and focused on the FITradeoff method.

For the applicability and relevance of this research, the nurses responsible for the triage process in the emergency care units were credited with a high relevance in the protocol selection, thus, a questionnaire was applied to the nurses of the four UPAs in order to know their opinions on the relevance of each subcriteria.

In the possession of this information, the data concerning the opinion of the nurses was taken to a specialist in the triage area. A second questionnaire was applied with this specialist questioning how good a protocol is for a criterion against a subcriterion.

With the data created by the specialist, a decision matrix was developed, which was systematized in the Excel file provided by FITradeoff software, and brought to the final decision maker.

In meetings with the final decision makers, it was made a ranking of importance of the criteria and then answered questions about preferences of the protocols, until finally the software select the most appropriate protocol according to the answers given.

## Results

### First step

#### Problematic

One of the most relevant points to the initial application of a multicriteria methodology is the definition of the central problem of the study. In this case, it will be a selection problem, because the main objective of the application is to select a specific protocol from one set, in others words, choose within a group of protocols the one that most meets the needs of UPAs.

#### Alternative

The alternatives presented in this research consist of the triage protocols studied, which are according to the literature the five most prestigious protocols in the world [17–20].

- A1 – Australasian Triage Scale (ATS)
- A2 – Canadian Triage and Acuity Scale (CTAS)
- A3 – Manchester Triage System (MTS)
- A4 – Emergency Severity Index (ESI)
- A5 – Spanish Triage System (SET)

#### Criteria

The evaluation criteria represent the guidelines to be used in the decision-making process, using them as characteristics of the triage protocols so that the alternatives of the problem can be analyzed from the perspective of the same guidelines.

They are commonly described as a goal to be achieved, and may, more formally, be presented in an objective function format. A set of criteria is knowledge as a family of criteria, which has priority to cover all objectives and ensure that there are no redundancies [36].

For this research it will be used not only criteria such as subcriteria, which are basically the smaller hierarchical parts of the model. This is a way of unraveling and facilitating the evaluation, giving greater certainty about the decisions taken [47].

This study was initially structured on four criteria, and twenty-nine subcriteria were taken to the final decision-makers for their validation, and the criteria not approved by the decision group were excluded. After validation by final decision makers, twenty-seven subcriteria and four criteria remained (Table 2).

**Table 2:** Final Criteria

Criteria	Subcriteria
Guidelines	Adult
	Pediatric
	Senior
	Pregnant
	Disabled
	Aggressive
	Alcoholics
	Nurses Attendance in Simple Cases
Ease of Evaluation	Medical History
	Pain Scale.
	Use of Medications
	Allergies
	Physical Evaluation
	Mental Evaluation
	Vital Signs
	Re-triage
Ease of Use	Use of Color Scale
	Maximum Waiting Time
	Maximum Triage Time
	Maximum Service Time
	Total Triage (5 levels)
	Partial Triage (2 + 3 levels)
	Nurses Attendance in Simple Cases
	Use of Computer and Software
Ease of Implementation	Training of employees
	Need for computer
	Use of specific software

### Second step

#### Analysis nurses

In order to insert into the data the experience and expertise of the nurses of Emergency Care Units, was established with decision makers, the relevance to know the opinion of nurses. Ensuring that the final decision contains not only the vision of the director of the unit, but of those professionals who perform and use the triage protocols daily.

For that, a questionnaire was applied, so that the nursing professionals of the units gave their opinions in front of three of the four evaluation criteria, these three because they represent actions that directly involve the triage process.

The questionnaire had a five-point Likert scale, which was developed in 1932 by the Ph.D. in psychology from Columbia University, Rensis Likert. This scale stood out for being able to identify more information than the models of the time, and became one of the most used in the world for its simplicity and ease of application and understanding by the interviewees [48, 49].

After the questionnaire was applied to the nurses, the aggregation process was performed, which is used when a decision is made in a group, that is, more than one person, and they do not reach an agreement or cannot meet. Thus, the aggregation is done, which can take place for the sum of values, or the mean of these [50, 51].

The subcriteria in the Guidelines, Ease of Evaluation and Ease of Use criteria were evaluated, being applied with five nurses from each of the four units, totaling twenty nurses. Thus, it is possible to observe the results in the questionnaire in Table 3.

**Table 3:** Nurses questionnaire data

Criteria	Subcriteria	UPAs
Guidelines	Adult	4,90
	Pediatric	4,10
	Senior	4,65
	Pregnant	3,60
	Disabled	3,95
	Aggressive	3,75
	Alcoholics	3,75
	Nurses Attendance in Simple Cases	3,45
Ease of Evaluation	Medical History	4,90
	Pain Scale.	4,55
	Use of Medications	4,55
	Allergies	4,95
	Physical Evaluation	4,40
	Mental Evaluation	4,40
	Vital Signs	4,95
	Re-triage	4,65
Ease of Use	Use of Color Scale	4,65
	Maximum Waiting Time	3,75
	Maximum Triage Time	3,90
	Maximum Service Time	4,30
	Total Triage (5 levels)	2,60
	Partial Triage (2 + 3 levels)	4,40
	Nurses Attendance in Simple Cases	4,00
	Use of Computer and Software	4,60

#### Analysis specialist

One of the most relevant steps for applying multicriteria decision analysis is the evaluation of the criteria. For this research will be used the analysis with specialists, is that uses one or more specialist of the studied area, that knows all the alternatives applied, and that can be able to evaluate the relations between the criteria, subcriteria and alternatives.

Starting from this point, a questionnaire was applied to the specialist, where s/he evaluated how well a subcriteria, belonging to a criterion, is an alternative. In other words, s/he analyzed how good a subcriteria is within the alternative.

It is important to point out that the research specialist works in one of the four UPA units, and has worked for many years in the triage process, from large hospitals and UPAs units. S/He has done specializations and studies in the triage area, and has knowledge of protocols such as the ones experienced at the units. Hence, s/he was considered by the authors and the final decision makers to be able to perform the role of specialist in the present research.

In the questionnaire, a five-point Likert scale was used, ranging from poor to excellent. Finally, an aggregation process was performed to determine the final value of each criterion with respect to each alternative, as shown in Table 4.

**Table 4:** Analysis Specialist

Criteria	Subcriteria	ATS	CTAS	MTS	ESI	SET
Guidelines	Adult	5	5	5	5	5
	Pediatric	5	5	1	5	0
	Senior	5	0	1	0	0
	Pregnant	5	3	0	5	0
	Disabled	0	2	2	0	0
	Aggressive	5	0	2	0	0
	Alcoholics	0	0	2	0	0
	Nurses Attendance in Simple Cases	0	0	0	0	5
Ease of Evaluation	Medical History	5	5	5	5	5
	Pain Scale.	5	5	5	0	5
	Use of Medications	0	5	0	0	0
	Allergies	0	5	0	0	0
	Physical Evaluation	4	4	0	0	0
	Mental Evaluation	4	0	0	0	0
	Vital Signs	1	0	0	0	5
	Re-triage	5	5	0	0	0
Ease of Use	Use of Color Scale	5	5	5	5	5
	Maximum Waiting Time	4	4	0	0	0
	Maximum Triage Time	0	0	4	4	4
	Maximum Service Time	5	5	5	0	5
	Total Triage (5 levels)	3	0	3	0	3
	Partial Triage (2 + 3 levels)	0	4	0	4	0
	Nurses Attendance in Simple Cases	0	0	0	0	4
	Use of Computer and Software	0	2	5	0	5
Ease of Implementation	Training of employees	5	5	5	5	5
	Need for computer	0	3	3	0	3
	Use of specific software	0	0	4	0	4

Table 4 presents the values given by the specialist, based on the protocols and the opinion of the nurses. Table 5 shows the result of the aggregation process.

**Table 5:** Aggregation

	ATS	CTAS	MTS	ESI	SET
Guidelines	25	15	13	15	10
Ease of Evaluation	24	29	10	5	15
Ease of Use	17	20	22	13	26
Ease of Implementation	5	8	12	5	12

#### Application and analysis of results

With the creation of the matrix (Table 5), which limits the weight space for each of the situations, it is possible to start the application in the FITradeoff software, which will initially request the order of preference of the decision maker in relation to the criteria, followed by a analysis for the removal of dominated alternatives.

**Fig. 3:**
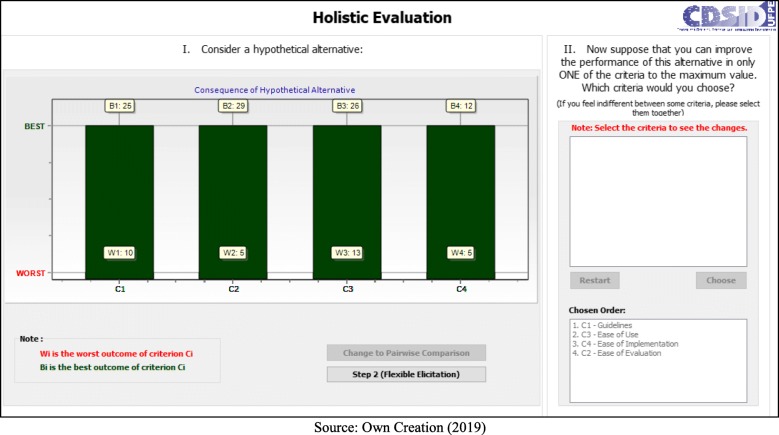
Elicitation of Criteria

In the process of eliciting the criteria, the decision makers chose: Guidelines> Ease of Use> Ease of Implementation> Ease of Evaluation. This choice can best be seen in Fig. 3.

From this process, two alternatives were dominated and excluded from the possible solutions; thus leaving the ATS, MTS and SET protocols, as observed in Table 6.

**Table 6:** Possible solutions

	K (Guidelines)	K (Ease of Use)	K (Ease of Implementation)	K (Ease of Evaluation)	Maximum Value
ATS	1	0	0	0	1
MTS	0,3684	0,3158	0,3158	0	0,6316
SET	0,3333	0,3333	0,3333	0	0,6667
	K (Guidelines)	K (Ease of Use)	K (Ease of Implementation)	K (Ease of Evaluation)	
Maximum Limit	1	0,5	0,333,333	0,25	
Minimum Limit	0,25	0	0	0	

Table 6 shows the values of the limits and weights for the possible solutions after the criteria elicitation process, which generates a reduction of the weight space. This reduction is a facilitator of the decision, because at that moment, the final decision maker may decide to take a solution from his own experience and knowledge in the area [41, 52].

**Fig. 4:**
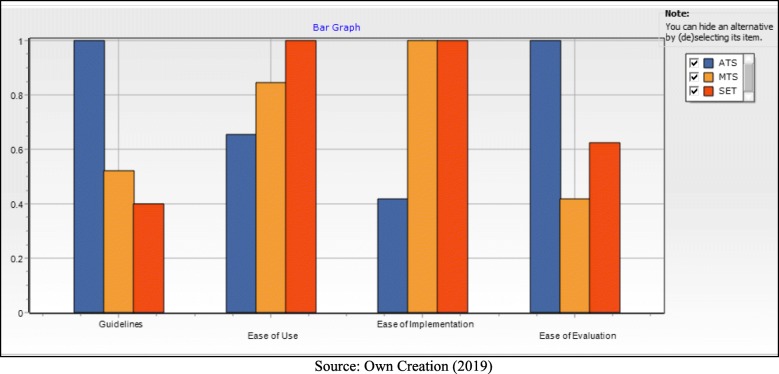
Bar Grafh

Based on these three possible solutions, FITradeoff presented the graphs (Fig. 4), in order to facilitate the final decision makers’ assessment, where they analyzed and decided not to have enough confidence to decide between the three possible solutions.

In Fig. 4 shows the bar graph, it compares the possible solutions, being easily observable which solution stands out in each one of the criteria. In this case, we have the ATS standing out in the Ease of Evaluation and Guidelines, and the SET protocol stands out in Ease of Use and Ease of Implementation tied with the MTS.

**Fig. 5:**
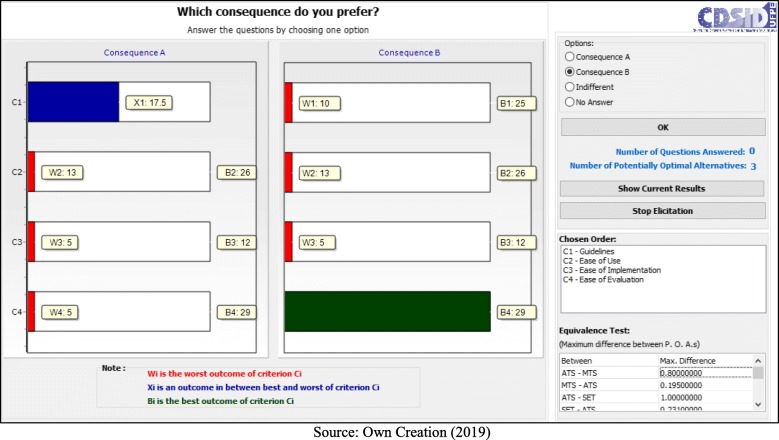
Question 1

**Fig. 6:**
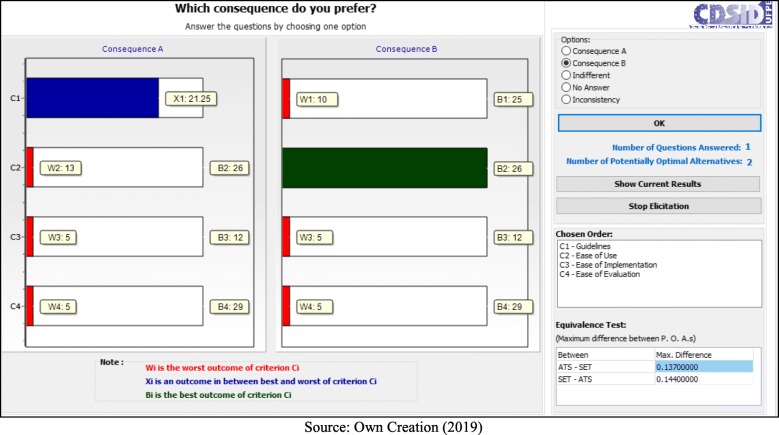
Question 2

**Fig. 7:**
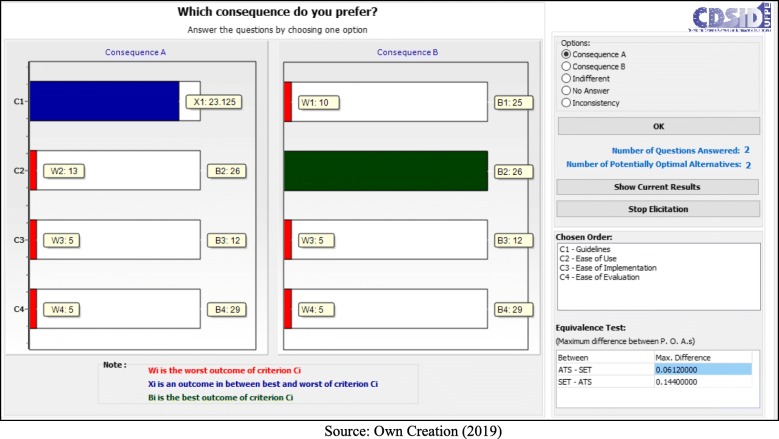
Question 3

**Fig. 8:**
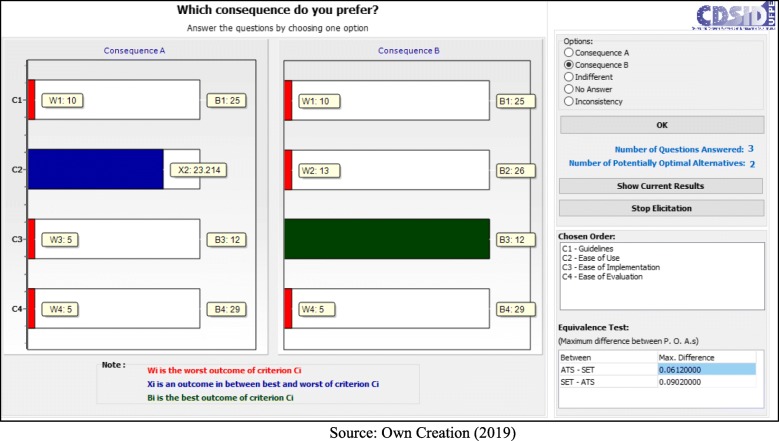
Question 4

**Fig. 9:**
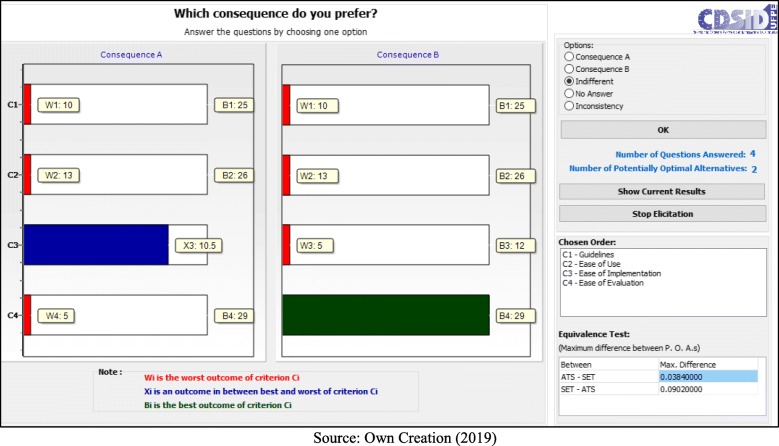
Question 5

**Fig. 10:**
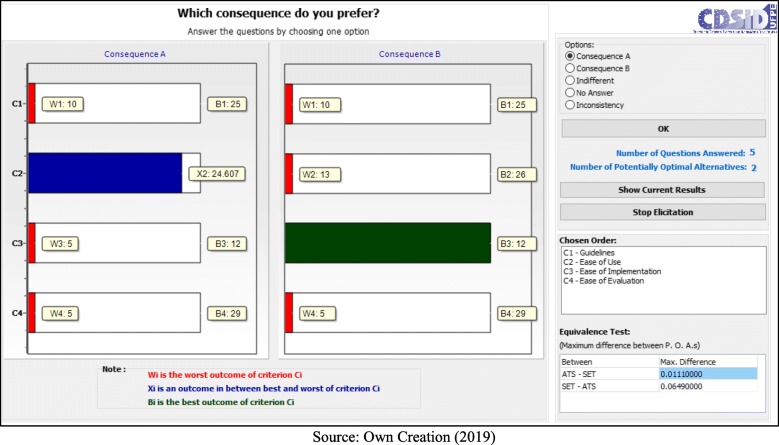
Question 6

**Fig. 11:**
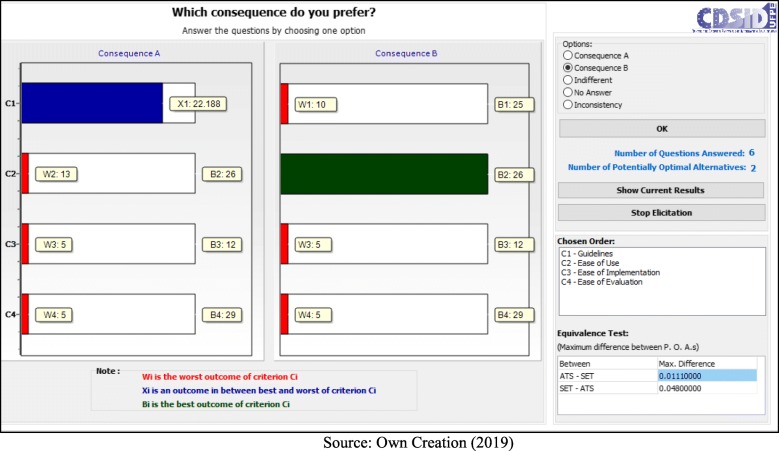
Question 7

Following the application process, the decision makers answered seven questions:

- C1 (Guidelines) < C4 (Ease of Evaluation) = B (Fig. 5);
– C1 (Guidelines) < C2 (Ease of Use) = B (Fig. 6);
– C1 (Guidelines) < C2 (Ease of Use) = A (Fig. 7);
– C2 (Ease of Use) < C3 (Ease of Implementation) = B (Fig. 8);
– C3 (Ease of Implementation) < C4 (Ease of Evaluation) = Indifferent (Fig. 9).
– C2 (Ease of Use) < C3 (Ease of Implementation) = A (Fig. 10);
– C1 (Guidelines) < C2 (Ease of Use) = B (Fig. 11).

Finally, the best solution found considering all units of UPAs in the city of Natal, was Alternative 5, the Spanish Triage System (SET). This solution can be better observed in Table 7, where the data of the weights and limits of the solution are demonstrated.

**Table 7:** Final Solution

	K (Guidelines)	K (Ease of Use)	K (Ease of Implementation)	K (Ease of Evaluation)	Maximum Value
SET	0,3058	0,2676	0,2389	0,1877	0,5847
	K (Guidelines)	K (Ease of Use)	K (Ease of Implementation)	K (Ease of Evaluation)	
Maximum Limit	0,338,697	0,282,014	0,238,908	0,187,713	
Minimum Limit	0,305,802	0,261,427	0,216,222	0,169,889	

**Fig. 12:**
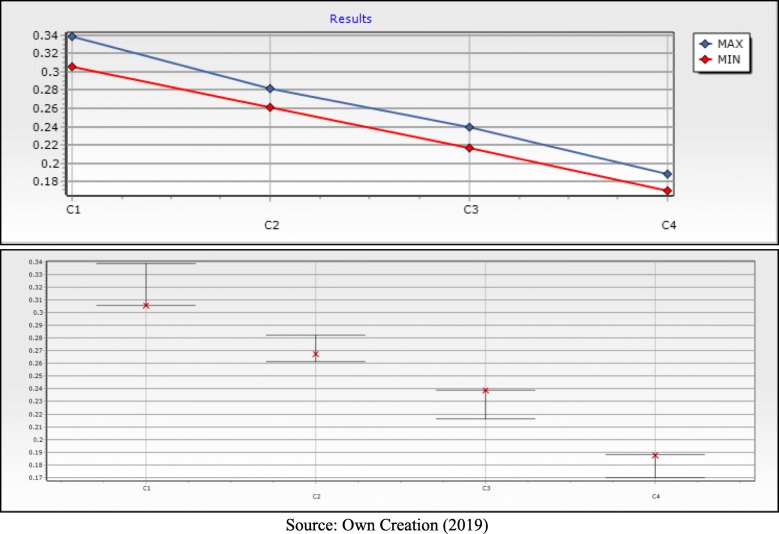
Limits of Weights

Figure 12 present graphically the values of the limits that were demonstrated in Table 7, these limits visually demonstrate the solution space of the response, in other words, it present the space generated from the answers given by the decision makers, creating the weights for each criteria and limiting the space until reaching the best possible solution.

## Discussion

Currently, the UPAs in Natal, Brazil have no triage system already structured and predetermined to be applied in all the units. This situation generates a difference in attendance and may lead to misclassifications. So, the question arises, how to choose the most appropriate protocol for UPAs?

Therefore, it is up to the manager of the health units to determine the protocol to be implemented, however this service is often complex and requires a lot of responsibility and knowledge of the manager. Thus, there are several tools capable of assisting the decision maker, and one of the best known are the multicriteria methods (MCDA), which help in the analysis of alternatives, based on conflicting criteria [36, 53].

In this situation, this study proposes to analyze the Emergency Care Units of the city of Natal-Brazil, and determine the most appropriate triage protocol for the units, using the multicriteria method known as FITradeoff.

In order to obtain the data, one has the definition of the problem, which for the study was problematic of choice, that is, to choose the most appropriate protocol among a series of alternatives.

Another point is the selection of the alternatives, which consists of the five famous protocols, which were previously determined.

Finally, the selection of the evaluation criteria, which for this study were divided into four criteria and twenty-nine subcriteria, through the process of validation with the decision makers, ended this stage with four criteria and twenty-seven subcriteria.

With the application made, it was possible to reach the main objective of the study, to determine the most appropriate protocol. The software was applied jointly in the units, obtaining as final solution the most suitable protocol for application in the UPAs of Natal-Brazil is the Spanish Triage System.

However, this research, besides indicating the most adequate protocol for the UPAs, also signaled a series of possibilities for the future, such as the study for the development of a specific protocol for UPAs. This one that would fit in the Brazilian reality and that could integrate in its system the specific characteristics of this type of medical unit.

Moreover, from the elaboration of a new triage protocol, it would be possible to develop specific software, which would aid in the integration of the UPA system, providing greater communication and interaction between the units.

## Conclusion

Relative to this research, reached its main objective, and determined that the Spanish Triage System is the most appropriate for the reality of UPAs.

It is expected that the new protocol can be implemented in the units and that this brings not only a standardization in the triage service, but a significant improvement in the service of general form, reducing the disorders caused by the overcrowding of the system.

Finally, it was concluded that the research reached its objectives, and in addition, opened the door to other studies, referring to a new protocol and software, moreover, added value both academically and to society in general, providing a more efficient service and reducing overcrowding.

## Data Availability

Data sharing is not applicable to this article as no datasets were generated or analysed during the current study.

## Abbreviations

ATS: Australian Triage Scale
CTAS: Canadian Triage Acuity Scale
ESI: Emergency Severity Index
MCDA: Multicriteria Decision Analysis
MTS: Manchester Triage System
PNAU: National Politic of Attention to Emergencies and Urgencies
SET: Spanish Triage System
SUS: Brazilian Unified Health System
UPA: Emergency Care Units
